# Inactivated alpha toxin from *Clostridium novyi* type B in nano-emulsion protect partially protects Swiss mice from lethal alpha toxin challenge

**DOI:** 10.1038/s41598-019-50683-2

**Published:** 2019-10-01

**Authors:** Mellanie Karoline C. Felix, Tullio T. Deusdará, Lucas Samuel S. Santos, Raimundo Wagner S. Aguiar, Roberto Franco T. Corrêa, Igor V. Brandi, Eliane M. Sobrinho, Bergmann M. Ribeiro, Luis André M. Mariúba, Paulo A. Nogueira, Kattyanne S. Costa, Kelvinson F. Viana, Alex Sander R. Cangussu

**Affiliations:** 1 Federal University of Tocantins – Postgraduate program in Biotechnology, Gurupi, TO Brazil; 2 Institute of Agrarian Science, Federal University of Minas Gerais, Montes Claros, MG Brazil; 3 Federal Institute of North Minas Gerais, Araçuaí, MG Brazil; 4 University of Brasília, Institute of Biological Sciences, Brasília, DF Brazil; 5 Institute Leônidas and Maria Deane – Fiocruz Amazônia, Manaus, AM Brazil; 6 Laboratory of Vaccine Technology – MSD Animal Health, Montes Claros, MG Brazil; 7 Federal Integrated University of Latin Americana – Laboratory of Molecular Biology and Biochemistry-ICVN, Foz do Iguaçu, PR Brazil

**Keywords:** Adjuvants, Nanofabrication and nanopatterning

## Abstract

Nano-emulsions are promising carriers for antigen delivery. Here, we evaluated the efficacy of a water-oil nano-emulsion containing concentrated, inactivated Clostridium novyi (C. novyi) type B supernatant culture (nano-iCnB) in protecting Swiss mice against a lethal dose of alpha toxin concentrated extract. Proteins were confirmed in the nano-iCnB and their stabilities were determined according physical parameters such as Zeta Potential (ZP). Biochemical, hematological parameters and morphological appearance of liver, spleen and thigh muscle alterations were examined to determine the safety of the compound. Partial protection against lethal doses was achieved in immunized mice despite low IgG titers. These data suggest that our nano-emulsion is a simple and efficient method of promoting antigen delivery for toxin-related diseases.

## Introduction

*C. novyi* type B is a strict anaerobic, endospore-forming, gram-positive bacterium. Its major virulence factor is an alpha toxin that causes necrotic hepatitis, known as black disease. Black disease symptoms include hemoglobinuria, reduced appetite, fever, lethargy, decreased milk production and blood-stained feces, all of which reduce productivity in sheep, horses, pigs and cattle^1,2^. Pathogenesis begins with spore germination in the liver, followed by development of vegetative cells and production of alpha toxin. Liver infection causes necrotic infarction with thick areas of hyperemia^1,3,4^. The acute disease is rapidly progressive and associated with a high mortality rate^5,6^.

Current vaccines are composed of multiple antigens adsorbed on aluminum hydroxide; nevertheless, their efficacies remain unsatisfying^4,7,8^. Aqueous adjuvant induces short-term responses, besides the fact that costs with handling and revaccination make them more expensive^9,10^. Thus, alternatives to reduce animal handling as well as elimination of interactions between antigens are necessary for vaccine development^10^. Antigens in nano-emulsions are promising alternatives to improve vaccine efficacy because they delay antigen delivery, generating good cellular and humoral responses for long periods^10,11^. Here, we demonstrated that our formulation is a substantial improvement and advance in terms of adsorption of antigens for control of clostridiosis.

## Results

### Physical characteristics analysis of the nano-iCnB

**Figure 1:**
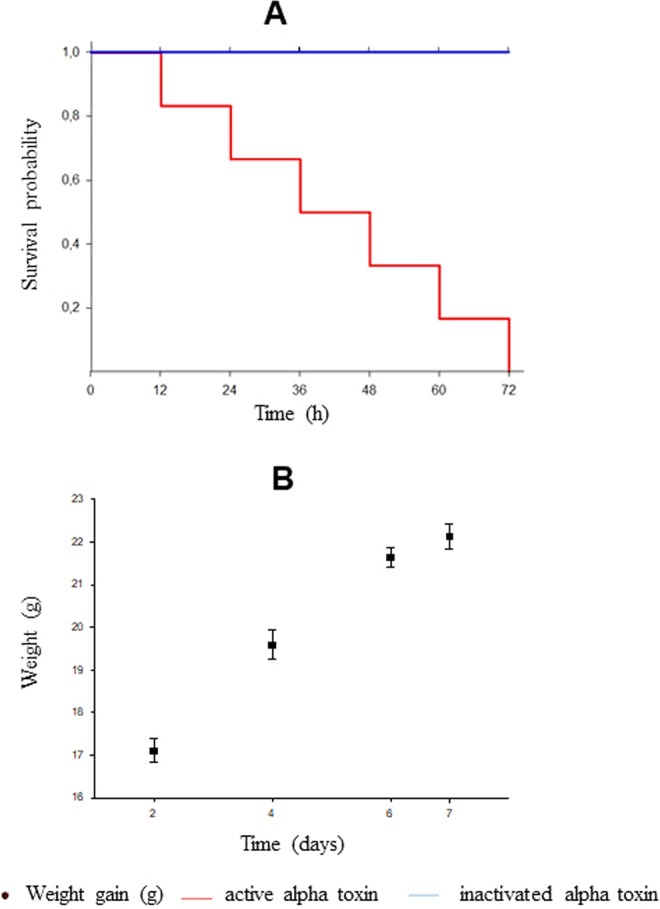
Determination of the lethal dose (LD_50_/mL) and inactivation process. (**A**) Survival probability of Swiss mice inoculated with active and inactivated alpha toxin. (**B**) Weight gain of Swiss mice inoculated with inactivated alpha toxin.

**Figure 2:**
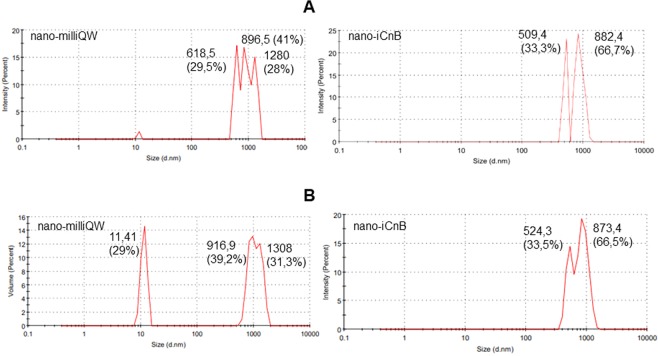
Physical characteristics of nano-iCnB and nano-milliQW nano-emulsion by Zetasizer Nano ZS90 Malvern Instruments. (**A**) Protein encapsulated droplets intensity. (**B**) Protein encapsulated droplet volume.

Alpha toxin-producing *C. novyi* cause gas gangrene, characterized by hemorrhage and muscle and soft tissue. After alpha toxin production, mice were inoculated to determine the lethal dose (LD_50_/mL) up to 72 hours (Fig. 1A). The inactivation process was confirmed by administration of inactivated alpha toxin for 7 days, noting absence of clinical signs and monitoring weight gain (Fig. 1B). The nano-iCnB was formulated in the 40% aqueous phase in a Vivaspin 6 MWCO 30000 at a final antigen concentration of 0.16 mg/ml, and 60% oil phase with ISA adjuvant. To assess stability of nano-iCnB from an ultrafiltrate of inactivated antigen, we compared the Zeta Potential (ZP) with antigen-free emulsion (nano-milliQW) consisting of 40/60% aqueous-oil phase (ISA adjuvant). The nano-milliQW had differentiated peaks with droplets of three diameters: 896.5, 618.5 and 1280 nm, suggesting that the system containing only adjuvant did not generate adequate size control. The intensity of 896.5 nm corresponded to 41% of the sample. The nano-iCnB revealed symmetrical peaks around of 509.4 and 882.4 nm, corresponding to 33.3% and 66.7% of the sample, respectively, confirming that proteins from nano-iCnB containing various sizes were adsorbed in nano-iCnB emulsion (Fig. 2A,B).

**Figure 3:**
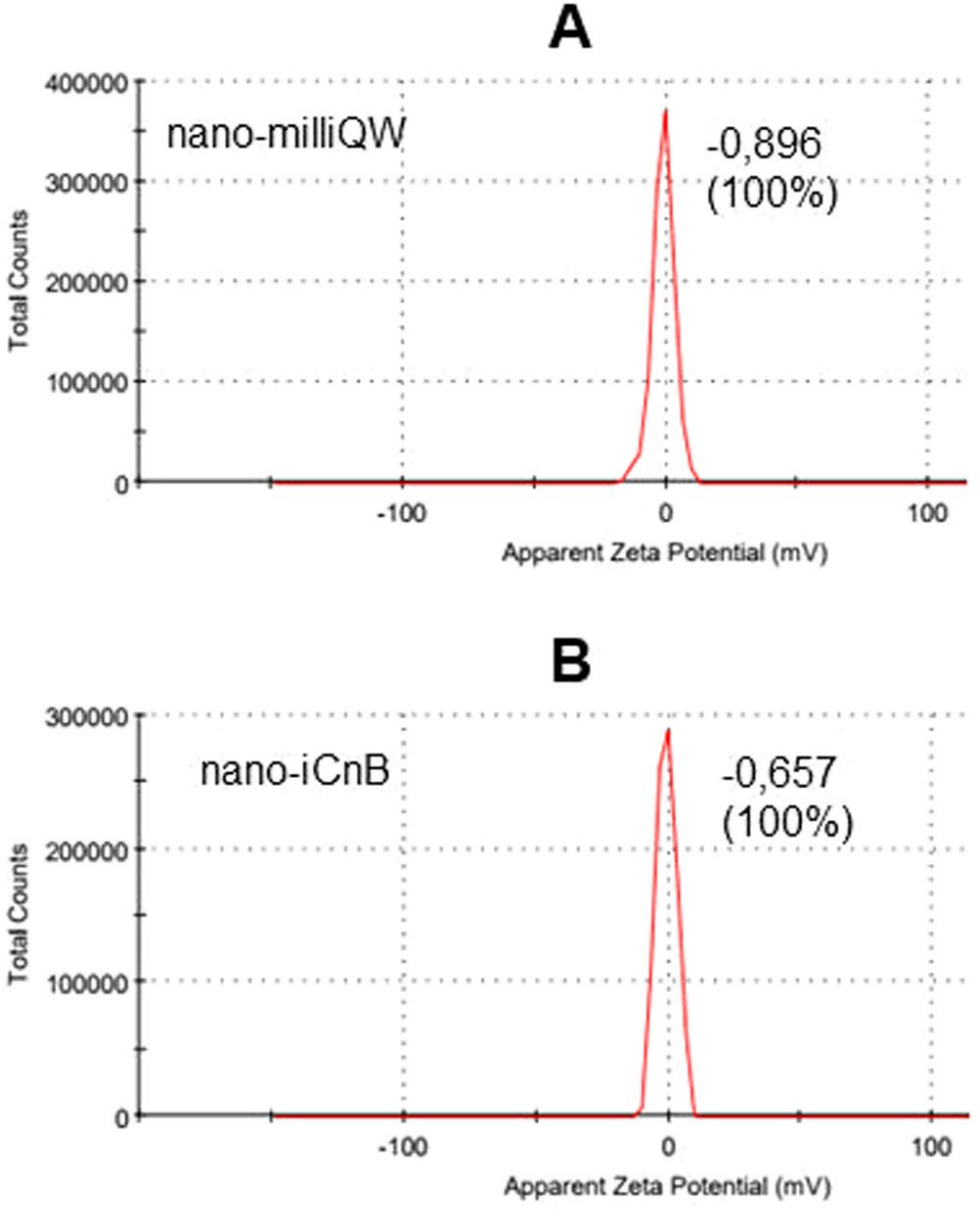
Physical characteristics of nano-iCnB and nano-milliQW nano-emulsion using Zetasizer Nano ZS90 Malvern Instruments. (**A**) Zeta Potential of nano-milliQW. (**B**) Zeta Potential of nano-iCnB.

Stability of the emulsion can be expressed according to ZP value, with higher ZP indicating lower stability on account of repulsive forces between charged particles that repel each other, overcoming the natural tendency of aggregation. The zeta potential of nano-iCnB was −0.657 mV, lower than nano-milliQW value of −0.896 mV, confirming its stability (Fig. 3A,B).

### Immunogenicity and efficacy test of the nano-iCnB

**Figure 4:**
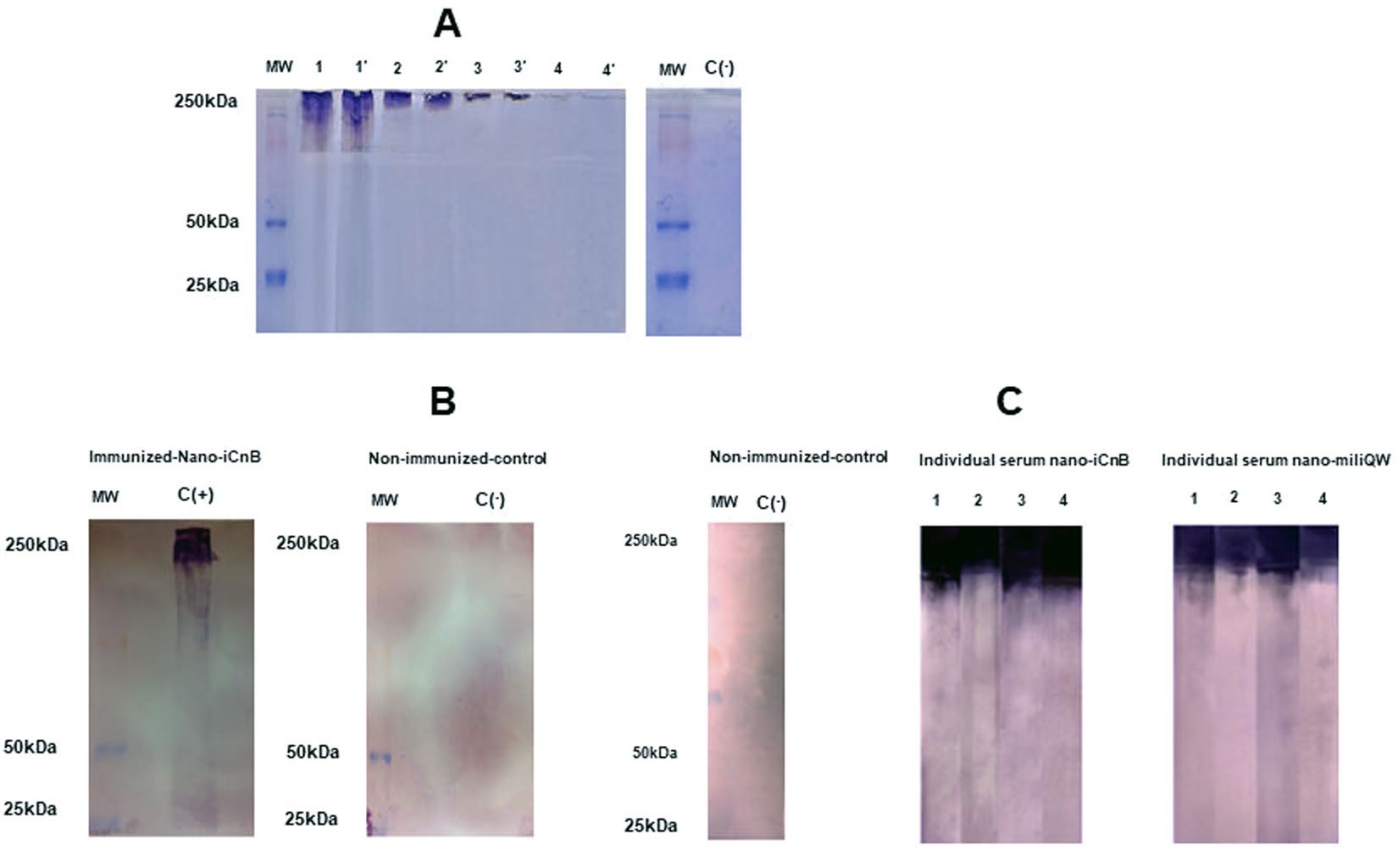
Antigenicity, immunogenicity and efficacy of nano-iCnB evaluated in Swiss mice. (**A**) Inactivated alpha toxin (>250 kDa) on 11% SDS-polyacrylamide gel electrophoresis. MW – Molecular weight. Line 1, 1′; 2, 2′; 3, 3′; 4, 4′, are representing dilution of inactivated alpha toxin: pure, 1:2; 1:4 and 1:8 in duplicate; respectively. (**B**) Immunoblotting of immunized group (nano-iCnB). (**C**) Immunoblotting of individual mice serum immunized with nano-iCnB and nano-milliQW.

Alpha toxin is a 250 kDa protein secreted in culture supernatants. We confirmed this using SDS/PAGE (Fig. 4A) and immunoblotting (Fig. 4B). Nano-iCnB´s ability to deliver antigen was demonstrated by detection of IgG in immunized swiss mice. Pooled sera of immunized animals reacted with the major antigen in the culture supernatants (Fig. 4B). We also determined antibody levels with reactivity of nano-iCnB individually. Individual mouse serum confirmed major reactivity with nano-iCnB antigen when compared with individual mouse serum of nano-milliQW or non-immunized mice (Fig. 4C). Pooled sera and individual mouse serum reacted specifically with the 250 kDa alpha toxin adsorbed in nano-iCnB indicating our nano-emulsion promoted antigen delivery (Fig. 4B,C).

### Safety of nano-iCnB according to liver function test and hematological data

**Figure 5:**
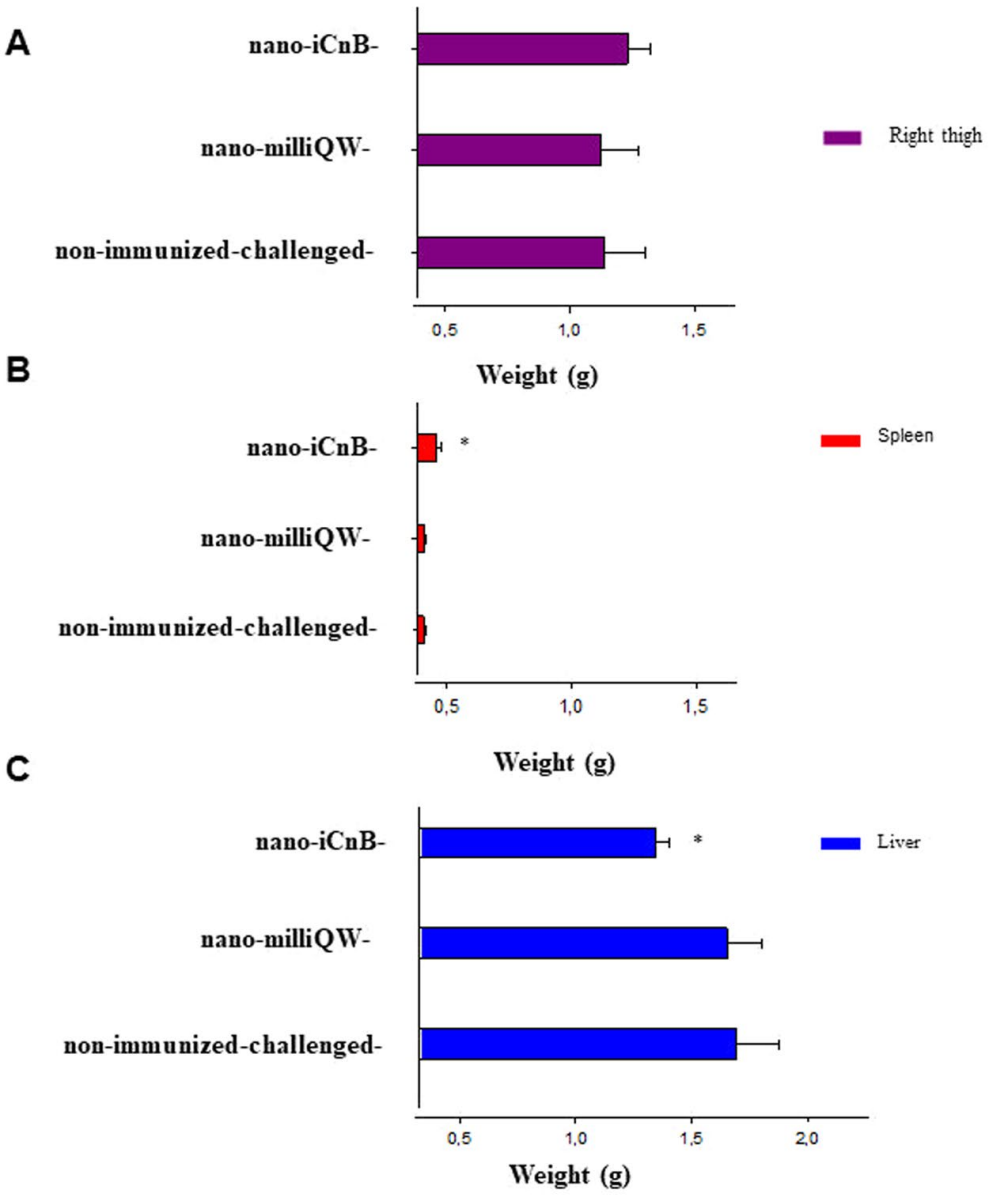
Safety test of immunized mice with nano-iCnB. (**A**) Morphological appearance of right thigh inoculated. (**B**,**C**) Spleen and liver of immunized and non-immunized-challenged mice as well as the nano-emulsion group. *p < 0.05 statistic differences.

The safety of nano-iCnB was demonstrated by absence of edema in the right thigh where the injection was performed (Fig. 5A). The spleens of nano-iCnB-immunized mice showed increased weight (Fig. 5B). Analysis of the variation of liver weight in these animals showed significant differences between the experimental groups. However, the nano-iCnB, nano-milliQW mice or non-immunized-challenged did not cause edema at the injection site (Fig. 5C). The blood samples collected from mice challenged with alpha toxin-concentrated extract before euthanasia were used to evaluate changes in hematological parameters and liver function (Table 1). These showed clinical pictures of leukocytosis, polycythemia, increased levels of alanine transaminase (AST), alanine transaminase (ALT) and alkaline phosphatase (AP) of non-immunized-challenged mice. Nano-iCnB immunized, Nano-milliQW or non-immunized-control mice showed normal values in all parameters and normal levels of AST, ALT and AP, while non immunized-challenged showed anemia.

**Table 1:** Hematologic data and liver function test of Swiss mice immunized with nano-iCnB.

Parameters	Nano-milliQW	Non-immunized-control	Nano-iCnB	Non-immunized-challenged	Reference
Total Leukocytes, (mm^3^)	9600 (864)^ns^	8466 (982)	11733 (499)	12933 (7128)	4300–10000, (mm^3^)
Hemoglobin, (g/dL)	14,73 (0,51)^ns^	10,60 (2,80)	16,17 (0,51)	20,73 (1,80)*	13–17, (g/dL)
Hematocrit, (%)	44,33 (1,53)^ns^	38,80 (4,50)	48,67 (1,53)	64,67 (3,05)*	39–51, (%)
Alanine Transaminase (ALT), (**U/L**)	38 (12)^ns^	32 (12)	31 (11)	211 (73)*	26–60, (U/L)
Aspartate aminotransferase (AST), (**U/L**)	130 (65)^ns^	156 (38)	180 (49)	414 (72)*	81–184, (U/L)
Alkaline phosphatase (AP), (**U/L**)	125 (74)^ns^	183 (7,20)	190 (6)	206 (28)	63–196, (U/L)

Non-immunized-control (healthy animals); nano-milliQW; nano-iCnB; Non-Immunized-challenged with 157 LD_50_.g^−1^. ns – not significant - sera from Swiss mice non-immunized-control vs sera from Swiss mice inoculated with nano-milliQW. *significant - hemoglobin (p < 0.05); hematocrit (p < 0.05); ALT (p < 0.04); AST (p < 0.01) sera from immunized Swiss mice vs Non-immunized-challenged.

### Partial protection of nano-iCnB

**Figure 6:**
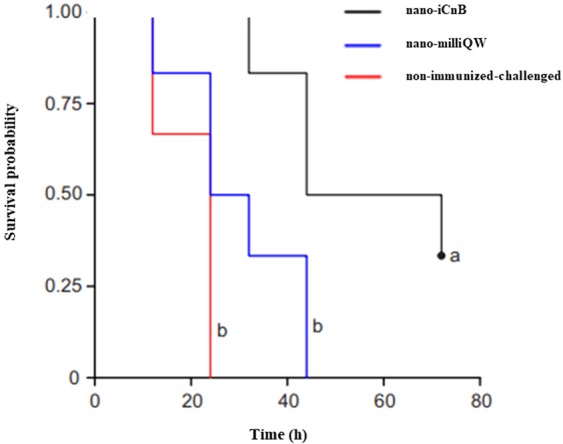
Survival curves of Swiss mice of experimental groups using the Kaplan-Meier method. The LT50 of the non-immunized-challenged group of 20.0 (15.1–24.9), while the LT50s of nano-milliQW and nano-iCnB were 30.0 (19.9–40.1) and 56.0 (40.8–71.2), respectively. The log rank statistic for the survival curves showed a significant difference between survival curves (p = 0.002). The survival curves were compared to the curves of the three groups (nano-milliQW, non-immunized-challenged and nano-iCnB) simultaneously, using TL50 as the comparison unit. Only animals in the nano iCnB group showed significant differences.

Kaplan-Meier survival curves showed that mice immunized with nano-iCnB had greater survival when challenged with a lethal dose of 157 LD_50_.g^−1^ than non-immunized-challenged or nano-milliQW mice (Fig. 6). The lethal dose generated symptoms in non-immunized-challenged and nano-milliQW mice, characterized by shaggy hairs, brown shading, rapid breathing, lacrimation, abdominal swelling and lethargy. Deaths started after 12 hours of the administration of challenge dose corresponding to 157 LD_50_.g^−1^ of alpha toxin. The LT_50_ of the non-immunized-challenged group of 20.0 (15.1–24.9), while the LT50s of nano-milliQW and nano-iCnB were 30.0 (19.9–40.1) and 56.0 (40.8–71.2), respectively. The log rank statistic for the survival curves showed a significant difference between survival curves (p = 0.002). The survival curves were compared to the curves of the three groups (nano-milliQW, non-immunized-challenged and nano-iCnB) simultaneously, using TL50 as the comparison unit. Only animals in the nano iCnB group showed significant differences.

## Discussion

Alpha toxin-producing *C. novyi* type B has substantial economic importance because of the damage it generates in livestock production chains. Here, we evaluated a nano-iCnB containing alpha toxin with 2178 amino acid residues, molecular weight ranging from 260 to 280 kDa^12,13^. We demonstrated partial protection in immunized Swiss mice using nano-iCnB, indicating its promising action as carriers for antigen delivery.

Critical reports regarding the use of aluminum hydroxide adjuvant for clostridial vaccines suggested that both size and conformational changes of toxoid affect surface-protein interactions with aluminum ligands during administration, thereby affecting the immune response^14^. New controlled release systems have been proposed as antigen delivery vehicles because of their utility as immunological adjuvants, with absence of toxicity, biodegradability, and slow release of encapsulated antigens^14^. Here, we observed that the nano-iCnB showed a high volume with 66.5% of the sample, suggesting improved encapsulation and controlled release of antigen (Fig. 2B).

The present study represents an innovative and simple technological strategy for the emulsification of inactivated toxins as immunogen^9,10^. Polymers have been used in bovine vaccine such as herpesvirus 1 (BHV-1), infectious bovine rhinotracheitis (IBR), and inactivated *C. botulinum* toxin with protection and safety being similar to that of the conventional vaccine^15–17^. As a proof of concept in this murine model, we consider it to be a relevant technological contribution of the use of nano-iCnB for immunization of Swiss mice. It represents an improvement in production of multiple antigens vaccines, specifically for the use of alpha toxin of *C. novyi* for control of hepatic necrosis.

Size, intensity and ZP are parameters used for analysis of interactions, transport of molecules and the availability of antigen at the immunization site^18^. Here, the nano-milliQW consisted of droplets of three diameters: 896.5, 618.5 and 1280 nm, suggesting that the system containing only the adjuvant did not produce adequate size control. However, the nano-iCnB showed only two bands: 509.4 and 882.4 nm with high volume, suggesting that the antigen helps to control droplet size in the nano-emulsion, giving rise to a fine emulsion^19,20^. The surface area to the volume in nanoemulsion can be affected by properties of the particles and their interaction with other systems. Small size particles show higher barrier properties for evaporation and increase the occlusion. These data suggest that the effect is affected by applied volume, particle size and crystallinity^21^.

ZP values are directly related to the stability of products, being obtained by the ratio of repulsion forces between charged particles that avoids the natural tendency to aggregate^22^. In nano-iCnB, the ZP was lower than that of nano-milliQW, demonstrating desirable physical characteristics for a W/O type emulsion vaccine formulation^20^. This suggests product stability, controlled release and stimulation of the immune response^18,23^.

Montanide ISA 61 VG is a new ready-to-use mineral oil-based adjuvant developed by SEPPIC Inc. (SEPPIC, France) being employed in the improvement of vaccines against FMD and BVDV virus^10,14^. Significant reduction of viremia (BVDV), even with a lower viral antigen concentration, was observed with use of vaccines formulated with ISA 61 VG. Here, for the first time, we used Montanide ISA 61 VG adjuvant together with bacterial antigen in nanoemulsion, and detected partial protection of Swiss mice after dose challenge (150 LD_50_g^−1^) with alpha toxin from *C novyi* tipe B. Immunized animals had later manifestations of the disease in some individuals, with a survival rate of 40%. Hepatocellular lesions and hepatocyte permeability may be indicated by changes in AST, ALT and cholestatic disorders caused by obstruction of the bile ducts, suggesting high metabolic activity in serum, as occurs in necrosis or disease in target tissues^24,25^. In contrast, nano-iCnB mice showed normal biochemical and hematological parameters. Furthermore, these animals did show signals of hypertrophy and congestion in liver. No edema in the right thigh nor with body or liver weight after 7 days of the final immunization indicated its safety. These data suggest that our nano-emulsion is a simple and efficient method that can be used to promote antigen delivery for toxin-related diseases.

This study had technical limitations as to improve the concentration of antigen present in nanoemulsion, as well to explore nano emulsion containing multiples antigen clostridial understanding your protective effect.

In conclusion, we proposed a simple and straightforward method of determining the effect of inactivated alpha toxin from *C. novyi* type B on a nanoemulsion. Nano-iCnB displayed safe and effective for stimulating immune responses in Swiss mice challenged with lethal doses of toxin.

## Materials and Methods

### Mouse lines and ethics in animal experimentation

Female Swiss mice (*Mus musculus*) weighing between 17 and 23 g were purchased from the Goiânia Central Goat Hospital - GO of the Federal University of Goiás (UFG). The animals were kept under conditions controlled for temperature, luminosity and odor. They were given feed (Biobase® - Brazil) and purified water ad libitum. The animals were maintained for a quarantine period of 7 days before the start of the experiments. The study was authorized by the Ethics and Research Committee of the Federal University of Tocantins (UFT) under number 23.101.006.832/2017-53 according to international recommendations for ethics in animal experimentation and by guidelines for animal use and care were based on the standards established by The Brazilian College of Animal Experimentation (COBEA).

### Anesthesia and euthanasia

Blood collection and morphological analyses of liver, spleen and inoculated right thighs were based on three animals per group at 0, 21 and 42 days. Prior to sample collection, the animals were anesthetized with ketamine 300 mg/Kg (Vetbrands, Brasil) and xylazine 22 mg/Kg (Vallée S/A) intraperitoneally and were euthanized by cervical dislocation to achieve rapid and painless death when mice showed intense lethargy, according to ethical protocols^26^. Immediately, cardiac puncture was performed and organs were collected before euthanasia at each time point for hematological and liver function tests, organ morphology and immunogenicity assays. During the challenge stage with active alpha toxin, animals in the experimental groups were anesthetized with ketamine and xylazine at the same concentration at the onset of clinical signs of infection.

### Bacterial lineage and culture conditions

*C. novyi* type B, virulent strain used for alpha toxin production and vaccinal antigen purchased from the NCTC (NCTC - National Collection Type cultures) was grown and stored in a culture bank with storage at the Laboratory of Biomolecules and Vaccines of the Federal University of Tocantins (LaBVac/UFT). Alpha toxin production was started in culture of *C. novyi* type B in Tarozzi culture medium and incubated at 37 ± 2 °C in BOD for 24 hours. Cultures were spun at 3000 *g* for 15 minutes to collect the supernatants. Vivaspin 6 MWCO 30000 membranes were used to concentrate inactivated alpha toxin used in W/O 40/60 nano-emulsions.

### Determination of lethal dose and the inactivation process of alpha toxin in mice

Toxicity of alpha toxin *C. novyi* type B was determined in Swiss mice by diluting the toxin from 1:4–1:16. From each dilution, 200 μL was administered intraperitoneally to four mice^27^. Determination of lethal dose (LD_50_/mL) was performed by observing the effect of the alpha toxin in the inoculated animals for 72 hours and quantifying by mathematical interpolation^28^. A lethal dose of 157DL_50_.g^−1^ was employed for challenge test (Fig. 1A).

Inactivation of alpha toxin in formaldehyde was performed at a ratio of 0.6% v/v at 37 °C under gentle agitation for 7 days. The inactivation test was performed with 200 µl intraperitoneal administration in three animals, followed by observation of the animals for 7 days^29^. Samples were considered inactivated if 100% of animals survived (Fig. 1B). An antigenic concentration of 0.16 mg/ml.g^−1^ was employed for the vaccine dose.

### W/O 40/60 nanoemulsion

In addition to LD_50_/mL, the total protein concentration of the antigen sample was determined using a Lab test®- total proteins 99–250 kit. Inactivated alpha toxin was emulsified in ISA adjuvant (MONTANIDE™ ISA 61 VG, Seppic, Brazil) to a final concentration of 0.16 mg/ml in the 40% aqueous phase and the 60% ISA adjuvant (oily phase). Nano-iCnB and nano-milliQW were prepared by passing the volume between sterile syringes connected by a silicone hose.

### Physical characteristics of the nano-iCnB

The physical characteristics of nano-iCnB were evaluated in the presence (culture supernatant) and water (nano-milliQW) of inactivated alpha toxin22. The diameter measurements of the emulsion droplets and zeta potential were performed using a Zetasizer Nano ZS90 from Malvern Instruments at 25 °C. Samples were diluted using ISA adjuvant at a ratio of 1:10.

The Dynamic Light Scattering (DLS) technique was used to determine the diameter of the droplets. In this technique, a beam of light is focused on the sample and droplet size is estimated from fluctuations in the intensity of light scattered at a given angle. The zeta potential is a measure of the magnitude of the static charges present at the interface between the droplets and the solvent. This parameter is important because it affects the stability of the droplets.

### Characterization of immunogenicity of nano-iCnB in swiss mice by immunoblotting

Characterization of the inactivated alpha toxin was performed using SDS/PAGE and antigenicity was evaluated by immunoblotting. We used 11% SDS/PAGE gels with buffer containing 1.5 M Tris-HCl pH 8.8 and 0.4% SDS. Electrophoresis buffer contained 25 mM Tris-HCl pH 8.3, 200 mM glycine and 1% SDS. The samples were heated to 100 °C for 5 minutes and were run at 70 Volts. The gels were stained with Coomassie blue for 12 h, and the protein bands were transferred to polyvinyl difluoride (PVDF) membranes using Trans-Blot® Turbo ™ Transfer System from Bio-Rad Lab and buffer with 5.82 g of tris base, 2.83 g glycine, 3.75 mL 10% SDS and 20% methanol, pH 9.2 for 60 minutes.

Electrotransfer was performed at 15 volts for 30 minutes. After transfer, the membranes were immersed in blocking solution with 1x PBS buffer + 3% milk powder, left overnight, and were subsequently washed in PBS 0.05%-Tween three times. Sera from healthy and diseased animals were diluted 1:2000 in 1x PBS + 0.5% BSA buffer and were applied to the membranes with gentle agitation for 60 minutes. Then, membranes were washed again for addition of alkaline phosphatase-conjugated anti-mouse at a dilution of 1:10000 under gentle agitation for 60 minutes. Membranes were again washed with PBS 0.05% Tween three times and once with alkaline phosphatase buffer under gentle agitation for 5 minutes. Membranes were submerged in NBT/BCIP substrate and the reaction was subsequently stopped. The membranes were washed with distilled water and dried in an oven.

### Experimental design

Female Swiss mice were separated into three groups (non-immunized, nano-emulsion and immunized) with 10 animals per group. In the immunized group, 100 µl of the nano-iCnB supernatant were administered at 0, 21, 42 days intramuscularly injected into the right thigh. Animals in the nano-emulsion and non-immunized groups were given 100 µl of PBS and nano-milliQW, respectively. The three experimental groups were monitored daily with weights measured and challenged with 157 LD_50_.g^−1^ after 7 days of the final immunization. Sick animals were anesthetized and euthanized and Kaplan-Meier survival curves were determined.

### Morphological appearance of liver, spleen and inoculated right thigh

Three animals were evaluated 7 days after the final immunization (day 42) to evaluate changes in liver, spleen and right thigh morphology. The degree of lesions at the site of administration was evaluated in terms of variation of organ weight, swelling, and redness as follows: 0) alterations absent; 1) discrete changes up to 25%; 2) moderate changes greater than 25%, but less than 50%; and 3) severe changes greater than 50%.

### Hematologic data and liver function tests

Seven days after the final immunization (day 42), experimental groups were challenged with a lethal dose of 157 LD_50_.g^−1^, followed by blood collection in the first 24 hours. The biological material was analyzed by the Veterinary Pet Shop Dog Center (Gurupi/TO - Brazil). Aspartate transaminase (AST), alanine transaminase (ALT), alkaline phosphatase (AP), leukograms, hemoglobin and hematocrit were determined. A group of three non-immunized animals were challenged with a lethal dose of 157 LD_50_.g^−1^ for the collection of sera from animals infected with active alpha toxin.

### Statistical analysis

The Kruskal-Wallis ANOVA test was used to verify the average and zeta-specific (ZS) sizes of the nano-iCnB and nano-milliQW. Morphological differences in liver, spleen, as well as liver and hematological data were compared between the immunized, nano-emulsion, non-immunized and sick animal groups (positive control) using data from three animals in each group. Significant differences were defined as p < 0.05 with 95% confidence intervals. Kaplan-Meier survival curves were used to display vaccine efficacy.

### Ethics approval and consent to participate

All experiments were performed in accordance with the ethical guidelines for experiments with mice, and the protocols were approved by the National Council for Control of Animal Experimentation, Federal University of Tocantins Animal Experimentation Committee (CEUA n° 23101.006.832/2017-53). The guidelines for animal use and care were based on the standards established by The Brazilian College of Animal Experimentation (COBEA).
